# Heme oxygenase-1 attenuates IL-1β induced alteration of anabolic and catabolic activities in intervertebral disc degeneration

**DOI:** 10.1038/srep21190

**Published:** 2016-02-15

**Authors:** Bo Hu, Changgui Shi, Chen Xu, Peng Cao, Ye Tian, Ying Zhang, Lianfu Deng, Huajiang Chen, Wen Yuan

**Affiliations:** 1 Department of Spinal Surgery, Changzheng Hospital, Second Military Medical University, No. 415 Feng Yang Rd, Shanghai 200003, China; 2 Shanghai Key Laboratory for Bone and Joint Diseases, Shanghai Institute of Orthopaedics and Traumatology, Shanghai Ruijin Hospital, Shanghai Jiao Tong University School of Medicine, China

## Abstract

Intervertebral disc degeneration (IDD) is characterized by disordered extracellular matrix (ECM) metabolism, implicating subdued anabolism and enhanced catabolic activities in the nucleus pulposus (NP) of discs. Pro-inflammatory cytokines such as interleukin-1β (IL-1β) are considered to be potent mediators of ECM breakdown. Hemeoxygenase-1 (HO-1) has been reported to participate in cellular anti-inflammatory processes. The purpose of this study was to investigate HO-1 modulation of ECM metabolism in human NP cells under IL-1β stimulation. Our results revealed that expression of HO-1 decreased considerably during IDD progression. Induction of HO-1 by cobalt protoporphyrin IX attenuated the inhibition of sulfate glycosaminoglycan and collagen type II (COL-II) synthesis and ameliorated the reduced expressions of aggrecan, COL-II, SOX-6 and SOX-9 mediated by IL-1β. Induction of HO-1 also reversed the effect of IL-1β on expression of the catabolic markers matrix metalloproteinases-1, 3, 9 and 13. This was combined with inhibition of the activation of mitogen-activated protein kinase signaling. These findings suggest that HO-1 might play a pivotal role in IDD, and that manipulating HO-1 expression might mitigate the impairment of ECM metabolism in NP, thus potentially offering a novel therapeutic approach to the treatment of IDD.

Low back pain (LBP) is a muscular disorder causing a severe social and economic burden. Investigators have shown that LBP is mainly associated with intervertebral disc degeneration (IDD)^1^. IDD is characterized by a series of pathogenic processes including cellular, biochemical and structural impairment, which result in a metabolic imbalance of the extracellular matrix (ECM)^2^. ECM metabolism disorder leads to the loss of water content, loss of the boundary between the outer annulus fibrosus (AF) and the inner nucleus pulposus (NP), and reductions in disc height and the capability to withstand mechanical load^3^, which are the main triggers of LBP.

NP cells play a pivotal role in ECM metabolism which can be divided into two aspects. With regard to anabolism, the major components of ECM are collagen type II (COL-II) and proteoglycans^4^. Aggrecan is the essential proteoglycan component of the intervertebral disc, with a large number of sulfated glycosaminoglycan (sGAG) side chains^5^; these molecules confer gel-like properties on the disc, enabling it to retain water and support a mechanical load^6^. The decreased expression of these matrix genes in IDD have been reported by investigators, indicating a reduction of anabolic activity^7^,^8^. On the catabolic side, previous studies demonstrated that catabolic proteinases such as matrix metalloproteinases (MMPs) and a disintegrin and metalloproteinase with thrombospondin motifs (ADAMTs) are upregulated in IDD, which is the main cause of ECM degradation in IDD progression^9^,^10^.

Pro-inflammatory cytokines play key roles in the pathophysiological processes of IDD^11^,^12^. Among them, interleukin-1β (IL-1β) is thought to be the predominant cytokine that is upregulated in degenerated discs, and this has led to its implication in increased production of MMPs and ADAMTs, and decreased synthesis of collagen type-II and aggrecan in NP cells^11^. Therefore, inhibition of the effect of IL-1β on ECM metabolism might facilitate ECM deposition and postpone the progression of IDD.

Heme oxygenase-1 (HO-1) breaks down heme to biliverdin, carbon monoxide (CO) and ferrous iron^13^. It has been reported that HO-1 can mediate cytoprotective effects under inflammatory conditions, and induction of HO-1 is thought to be an adaptive cellular response against stress^14^. A previous study showed that HO-1 induction reversed the actions of IL-1β on human chondrocyte ECM metabolism^15^, while Rousset and colleagues^16^ demonstrated that HO-1 regulates expression of MMP-1, MMP-13 and ADAMTS-4 in chondrocytes. The study also revealed that the effect of HO-1 on MMP-1 secretion may via its regulation of NADPH oxidases (NOX) 4 and reactive oxygen species (ROS) production in chondrocytes^16^. In IDD, it has been reported that high HO-1 expression can inhibit oxidative stress in an animal model of IDD^17^. However, the role of HO-1 in human IDD is still poorly understood. Based on these previous results, we hypothesized that HO-1 might regulate the alteration of ECM metabolism induced by the pro-inflammatory cytokine IL-1β in human NP cells. Hence, the purpose of the present study was to determine the relationship between HO-1 and IDD, and, moreover, to investigate whether HO-1 participates in anabolic and catabolic processes in human NP cells.

## Results

### Expression of HO-1 and its correlation with Pfirrmann grades in human nucleus pulposus tissues

**Figure 1:**
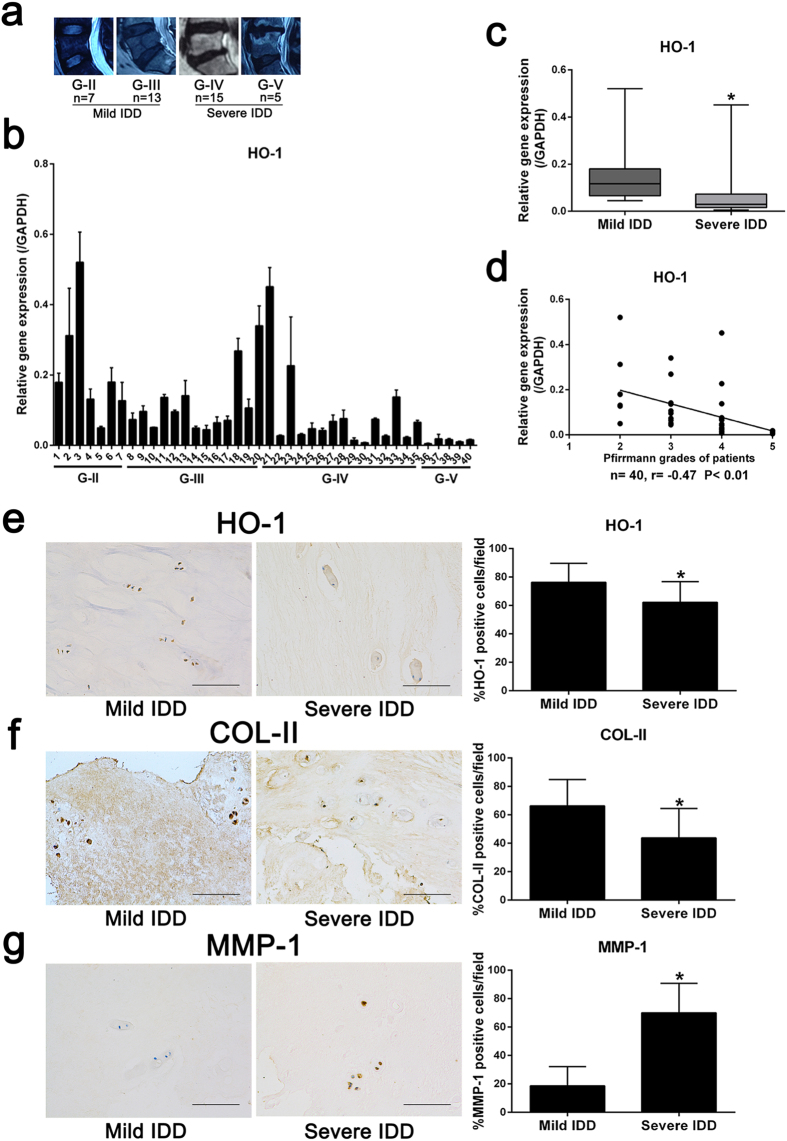
The expression of HO-1 in human nucleus pulposus tissues (**a**) Disc tissues from patients were collected and classified according to the modified Pfirrmann grading system, and samples of grade II/III (n = 20) were assigned to the mild IDD group, while samples of grade IV/V (n = 20) were allocated to the severe IDD group. (**b**) Real-time PCR of human degenerated intervertebral disc samples showed that the expression of HO-1 is consistently higher in the Grade II and Grade III (mild degeneration) samples than in Grade IV and Grade V (severe degeneration) samples. The results were normalized to the expression of GAPDH. (n = 40, mild IDD = 20, severe IDD = 20). (**c**) We further pooled the samples into two groups according to the clinical significance (mild and severe IDD), and the whisker box plot showed the HO-1 expression is considerably higher in mild than severe groups. (**d**) Gene expression of HO-1 was negatively correlated with the Pfirrmann grades of 40 human NP tissues (n = 40, r = −0.47). Immunohistochemistry of the human NP sections showed that numbers of HO-1 (**e**) and COL-II (**f**) -positive cells in the mild IDD group were remarkably decreased compared with those in the severe IDD group. The number of cells immunopositive for MMP-1 (**g**) were substantially higher in severe IDD compared with those in mild IDD NP tissues. Values represent the mean and standard deviation (SD). *P < 0.05, bars = 100 μm.

We estimated the degree of IDD by evaluating Magnetic Resonance Imaging (MRI) according to the modified Pfirrmann grading system (Fig. 1a). As a result, 20 NP samples with grade II and III IDD were categorized as the mild IDD group; for comparison, another 20 specimens with grade IV and V were set as the severe IDD group. Next, we performed real-time polymerase chain reaction (PCR) to measure the mRNA expression of HO-1 in NP from mild and severe IDD (Fig. 1b). The results showed that the mRNA expression of HO-1 in NP from severe IDD was significantly downregulated compared to those of NP from mild IDD (n = 40, *P* = 0.0004; Fig. 1c). We also found that the gene expression of HO-1 was negatively correlated with the Pfirrmann grades of 40 human NP tissues (n = 40, r = −0.47, *P* = 0.0023; Fig. 1d). Immunohistochemistry of human NP sections showed that the numbers of HO-1-positive cells were significantly decreased in the mild IDD group compared with the severe IDD group (*P* = 0.0031; Fig. 1e). Meanwhile, the numbers of cells immunopositive for COL-II and MMP-1 were significantly lower and higher, respectively, in severe IDD compared with mild IDD NP tissues (*P* = 0.0014 and *P* < 10^−4^, respectively; Fig. 1f,g).

### Induction of HO-1 by CoPP in human NP cells

**Figure 2:**
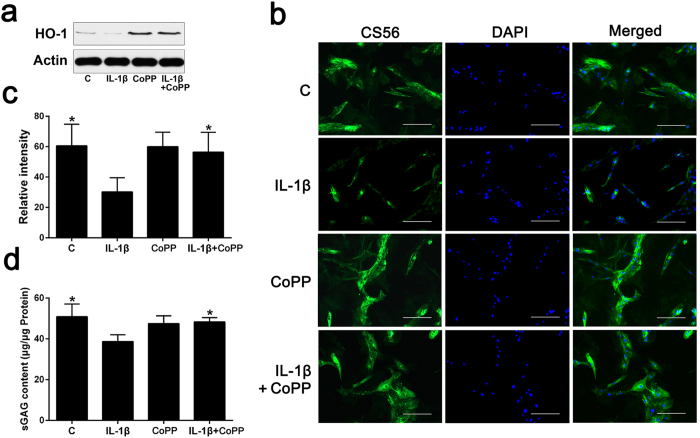
Effects of CoPP on HO-1 expression and sGAG synthesis in human NP cells (**a**) Western blot analysis revealed that HO-1 protein levels were remarkably increased by CoPP in the presence or absence of IL-1β. (**b,c**) Immunofluorescence microscopy showed that the immunostaining and relative immunofluorescence intensity of CS, the major component of sGAG, were lower after IL-1β stimulation and this effect was ameliorated by HO-1 induction (bars = 100 μm). (**d**) DMMB assay showed that the HO-1 inducer CoPP considerably rescued the loss of sGAG content caused by IL-1β without influencing basal sGAG synthesis. Values represent the mean and SD. *P < 0.05 with respect to IL-1β.

To determine the effects of cobalt protoporphyrin IX (CoPP) on HO-1 expression, human NP cells from IDD patients were treated with CoPP (10 μM) for 24 h as previously reported^15^. CoPP did not present substantial cytotoxicity at this concentration, as determined by cell counting kit-8 (98.74% ± 8.32% viability after 24 h incubation). The expression of HO-1 was examined by western blot analysis. We observed that HO-1 protein was remarkably increased by CoPP in the presence or absence of IL-1β (Fig. 2a). HO-1 small interfering RNA (siRNA) treatment attenuated the effects of CoPP on HO-1 expression compared with a negative control (NC) siRNA (Fig. 2a), indicating an effective knockdown of HO-1 following siRNA transfection in human NP cells.

### Reduction of glycosaminoglycan synthesis by IL-1β is ameliorated by HO-1 induction

In order to investigate the effect of HO-1 induction on sGAG synthesis under IL-1β stimulation, NP cells were treated with CoPP (10 μM) in the presence or absence of IL-1β. Immunofluorescence microscopy showed that the immunostaining and relative immunofluorescence intensity of chondroitin sulfate (CS) (Fig. 2b), which is the major component of sGAG, were lower after IL-1β stimulation and this effect was significantly ameliorated by HO-1 induction (*P* = 0.0025; Fig. 2c). The results of 1,9-dimethylmethylene blue (DMMB) assay revealed that the HO-1 inducer, CoPP, significantly rescued the reduction of sGAG content induced by IL-1β without influencing basal sGAG synthesis (*P* = 0.0138; Fig. 2d).

### IL-1β inhibition of collagen type II deposition is abolished by HO-1 induction

**Figure 3:**
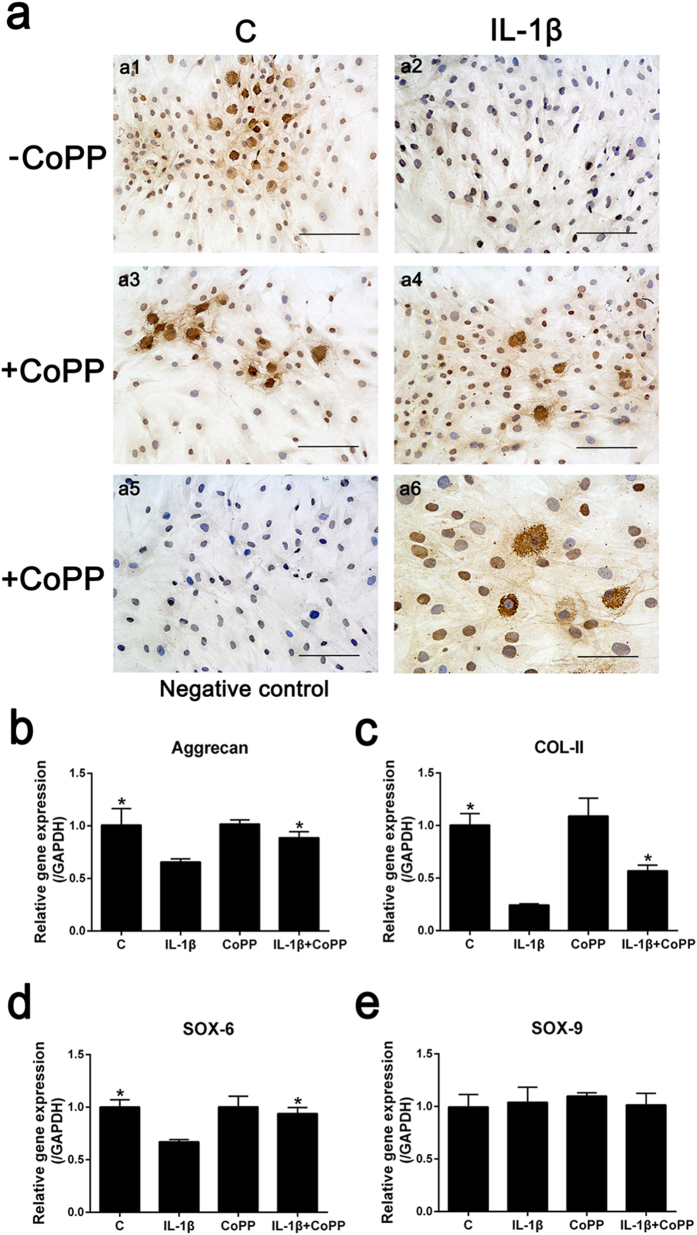
CoPP rescues ECM anabolic gene expression and collagen type II deposition in IL-1β-treated cells (**a**) Immunocytochemistry assay revealed that IL-1β treatment downregulated the expression of COL-II in human NP cells (a2). Stimulation with the HO-1 inducer CoPP partially abolished the effect of IL-1β and prevented the loss of COL-II deposition induced by IL-1β (a4,a6). Bars = 100 μm (a1–a5) or 50 μm (a6). Real-time PCR showed that IL-1β treatment led to a remarkable downregulation of gene expression of aggrecan (**b**), COL-II (**c**) and SOX-6 (**d**) in human NP cells, and addition of CoPP to the cells treated with IL-1β notably increased the gene expression of aggrecan, COL-II and SOX-6 although they did not reach basal levels. (**e**) Treatment with IL-1β did not affect the gene expression of SOX-9 in human NP cells. Values represent the mean and SD. *P < 0.05 with respect to IL-1β.

To confirm the effect of CoPP on COL-II deposition with IL-1β treatment, we conducted immunocytochemistry (Fig. 3a) and the results showed that IL-1β treatment downregulated the expression of COL-II in human NP cells (Fig. 3a2). However, stimulation with the HO-1 inducer, CoPP, partially abolished the effect of IL-1β and prevented the loss of COL-II deposition induced by IL-1β (Fig. 3a4,a6).

### Downregulation of ECM anabolic genes by IL-1β is attenuated by HO-1 induction

To further investigate the effect of HO-1 induction on ECM anabolic gene expression under the stimulation of IL-1β, we carried out real-time PCR to measure the gene expression of aggrecan, COL-II, SOX-6 and SOX-9. As previously reported^10^, we observed that IL-1β treatment led to a significantly downregulation of gene expression of aggrecan, COL-II and SOX-6 in human NP cells (*P* = 0.0185, *P* = 0.0003 and *P* = 0.0013, respectively; Fig. 3b–d) although it did not affect the expression of SOX-9 (*P* = 0.7077; Fig. 3e). Addition of CoPP to the cells treated with IL-1β significantly increased the gene expression of aggrecan, COL-II and SOX-6 although they did not return to basal levels (*P* = 0.0033, *P* = 0.0005 and *P* = 0.0015, respectively; Fig. 3b–d).

### HO-1 reverses IL-1β induction of ECM catabolic gene expression

**Figure 4:**
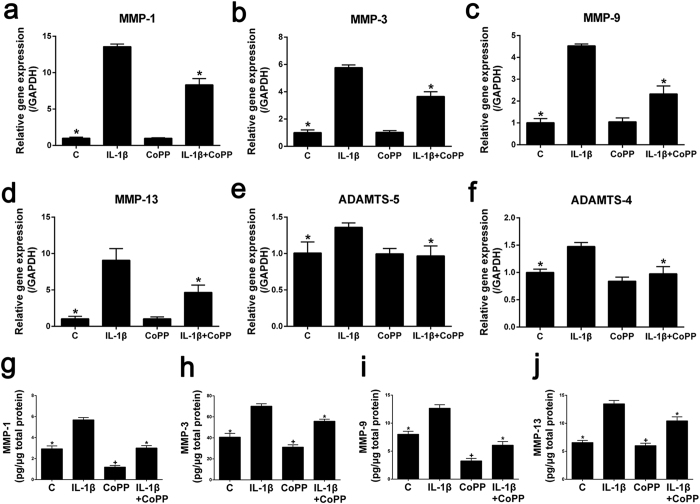
Effects of HO-1 induction by CoPP on expression of ECM catabolic genes in human NP cells Real-time PCR revealed that the induction of gene expression of the ECM catabolic enzymes MMP-1 (**a**), MMP-3 (**b**), MMP-9 (**c**), MMP-13 (**d**), ADAMTS-5 (**e**) and ADAMTS-4 (**f**) by IL-1β treatment was ameliorated by the HO-1 inducer CoPP. Treatment with CoPP alone did not affect the basal levels of expression of these catabolic genes (*P* > 0.05). ELISA showed that IL-1β stimulation of the protein levels of MMP-1 (**g**), MMP-3 (**h**), MMP-9 (**i**) and MMP-13 (**j**) were attenuated by induction of HO-1. The data also showed that treatment with CoPP alone downregulated the basal protein levels of MMP-1, MMP-3, MMP-9 and MMP-13. Values represent the mean and SD. *P < 0.05 with respect to IL-1β. ^+^*P* < 0.05 with respect to C.

Since IL-1β treatment has been reported to induce catabolic gene expression^10^, we used real-time PCR and enzyme-linked immunosorbent assay (ELISA) to assess whether HO-1 induction modulates IL-1β-induced catabolic gene expression in mRNA and protein level. As expected, gene expression of the ECM catabolic enzymes MMP-1, 3, 9, 13 and ADAMTS-4 and 5 were strongly increased by IL-1β treatment (Fig. 4a–f). Induction of HO-1 by CoPP significantly downregulated the gene expression of all these catabolic genes induced by IL-1β although they did not return to basal levels (*P* = 0.0006, *P* = 0.0008, *P* = 0.0005, *P* = 0.0156, *P* = 0.0103, *P* = 0.0045, respectively; Fig. 4a–f). Treatment with CoPP alone did not affect the basal levels of expression of these catabolic genes (Fig. 4a–f). Consistent with the results of real-time PCR, the protein levels of MMP-1, 3, 9, and 13 were all considerably increased by IL-1β treatment (Fig. 4g–j). Induction of HO-1 by CoPP significantly attenuated the IL-1β-mediated upregulation of MMP-1, 3, 9, and 13 protein levels in human NP cells (*P* < 10^−4^, *P* < 10^−4^, *P* < 10^−4^ and *P* < 10^−4^, respectively; Fig. 4g–j). Interestingly, treatment with CoPP alone significantly downregulated the basal protein levels of MMP-1, 3, 9, and 13 (*P* < 10^−4^, *P* < 10^−4^, *P* < 10^−4^ and *P* = 0.0135, respectively; Fig. 4g–j), which was not observed by real-time PCR analysis (Fig. 4a–d).

### The effect of CoPP on anabolic and catabolic activities induced by IL-1β mainly depend on HO-1 induction

**Figure 5:**
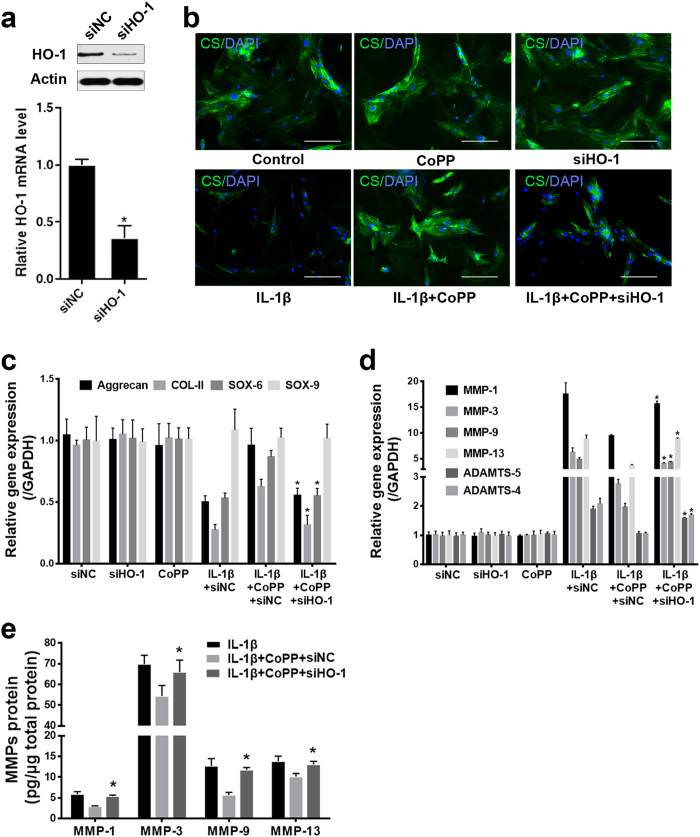
CoPP attenuates the effects of IL-1β on anabolic and catabolic activities mainly depend on HO-1 induction (**a**) Western blot and real-time PCR analysis revealed that HO-1 siRNA treatment inhibitied HO-1 expression compared with a scramble siRNA that targets none of the related genes as negative control. (**b**) si-HO-1 treatment attenuated the effect of CoPP on the inhibition of CS synthesis induced by IL-1β (bars = 100 μm). (**c**) HO-1 siRNA treatment substantially weakened the effect of CoPP on the down regulation of anabolic genes (Aggrecan, COL-II and SOX-6) induced by IL-1β. (**d,e**) HO-1 siRNA treatment considerably subsided the effect of CoPP on the increment of catabolic genes (MMP-1, MMP-3, MMP-9, MMP-13, ADAMTS-5 and ADAMTS-4) and proteins (MMP-1, MMP-3, MMP-9 and MMP-13) induced by IL-1β. Values represent the mean and SD. *P < 0.05 with respect to IL-1β+ CoPP+ siNC.

We further performed experiments with HO-1 siRNA to testify whether the effect of CoPP was depended on HO-1 induction. HO-1 siRNA transfection considerably down-regulated the expression of HO-1 (Fig. 5a), HO-1 siRNA treatment substantially attenuated the rescue effect of CoPP on the IL-1β induced down regulation of anabolic genes (Aggrecan, COL-II and SOX-6) and the inhibition of CS synthesis (Fig. 5b,c). The increment of catabolic genes (MMP-1, MMP-3, MMP-9, MMP-13, ADAMTS-5 and ADAMTS-4) and proteins (MMP-1, MMP-3, MMP-9 and MMP-13) induced by IL-1β was reversed by CoPP treatment, and this was abolished by HO-1 siRNA (Fig. 5d,e).

### Activation of MEK/ERK signaling by IL-1β is offset by HO-1 induction

**Figure 6:**
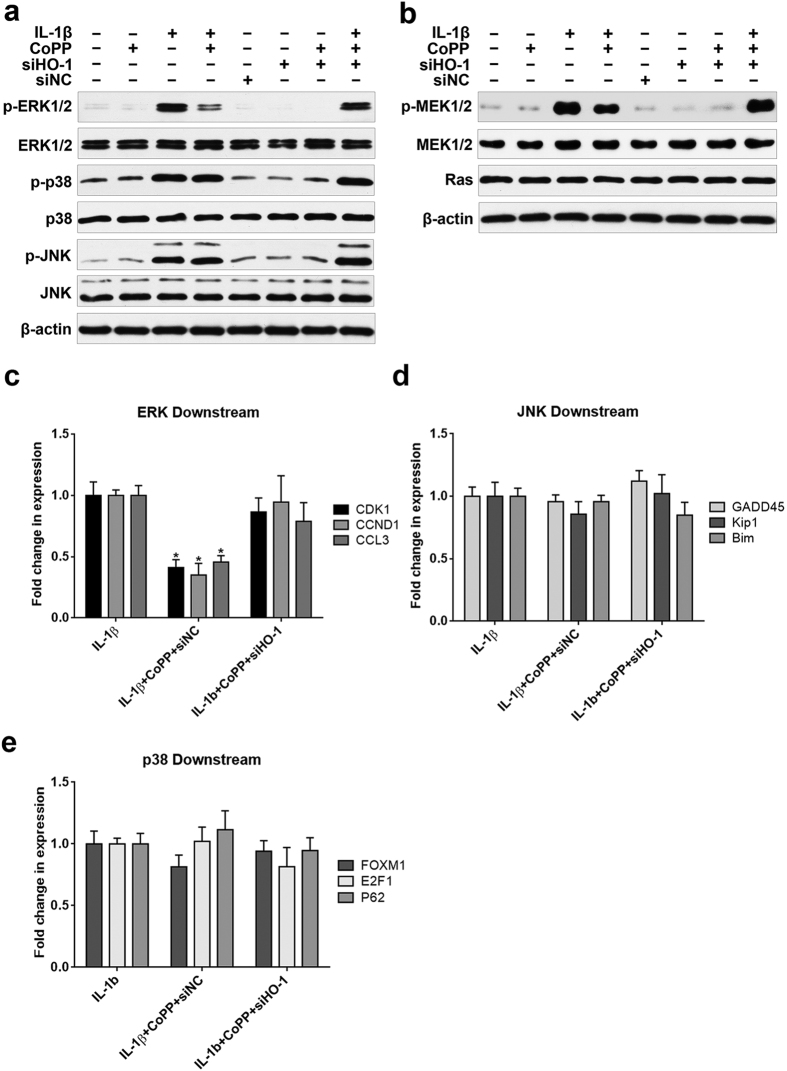
Effects of IL-1β and HO-1 regulation on MAPK signaling (**a**) Western blot analysis showed that the activation of ERK1/2 by IL-1β was remarkably weakened by HO-1 induction in human NP cells, whereas HO-1 siRNA treatment abolished this effect. (**b**) Treatment with CoPP attenuated the activation of MEK1/2 by IL-1β, while the change of Ras level cannot be observed. (**c**) The gene expression of ERK1/2 downstream transcriptional targets (CDK1, CCL3 and CCND1) was reduced by CoPP under IL-1β stimulation, (**d,e**) while CoPP cannot affect the expression of JNK and p38 downstream molecules (GADD45, Kip1, Bim, FOXM1, E2F1 and p62) induced by IL-1β. *P < 0.05 with respect to IL-1β.

Mitogen-activated protein kinase (MAPK) signaling has been reported to be activated by IL-1β in many cell types^18^,^19^,^20^, and investigators have previously demonstrated that extracellular signal-regulated kinase 1/2 (ERK1/2), p38 and c-Jun N-terminal kinase (JNK) are involved in the ECM anabolism and catabolism of NP cells^21^,^22^,^23^,^24^,^25^,^26^. Therefore, we used western blot analysis to examine whether HO-1 induction modulates this signaling pathway. The results showed that activation of ERK1/2 by IL-1β was remarkably weakened by HO-1 induction in human NP cells, whereas HO-1 siRNA treatment abolished this effect (Fig. 6a), however, CoPP treatment did not affect p38 and JNK activation by IL-1β stimulation (Fig. 6a). To further identify the effect of HO-1 on upstream regulators of ERK1/2, we examined whether HO-1 induction affected typical Ras/MEK/ERK pathway. The results of western blot analysis showed that induction of HO-1 by CoPP attenuated the activation of MEK1/2 by IL-1β, while the change of Ras level cannot be detected (Fig. 6b). To further validate the result, we found that the expressions of downstream transcriptional targets of ERK1/2, including Cyclin-dependent protein kinase 1 (CDK1), CYCLIN D1 (CCND1) and CCL3, were reversed by CoPP treatment under IL-1β stimulation, while the level of downstream molecules of JNK (GADD45, Kip1 and Bim) and p38 (FOXM1, E2F1 and p62) has not been affected (Fig. 6c–e).

### The effect of HO-1 induction is mediated via the regulation of NOX/ROS system

**Figure 7:**
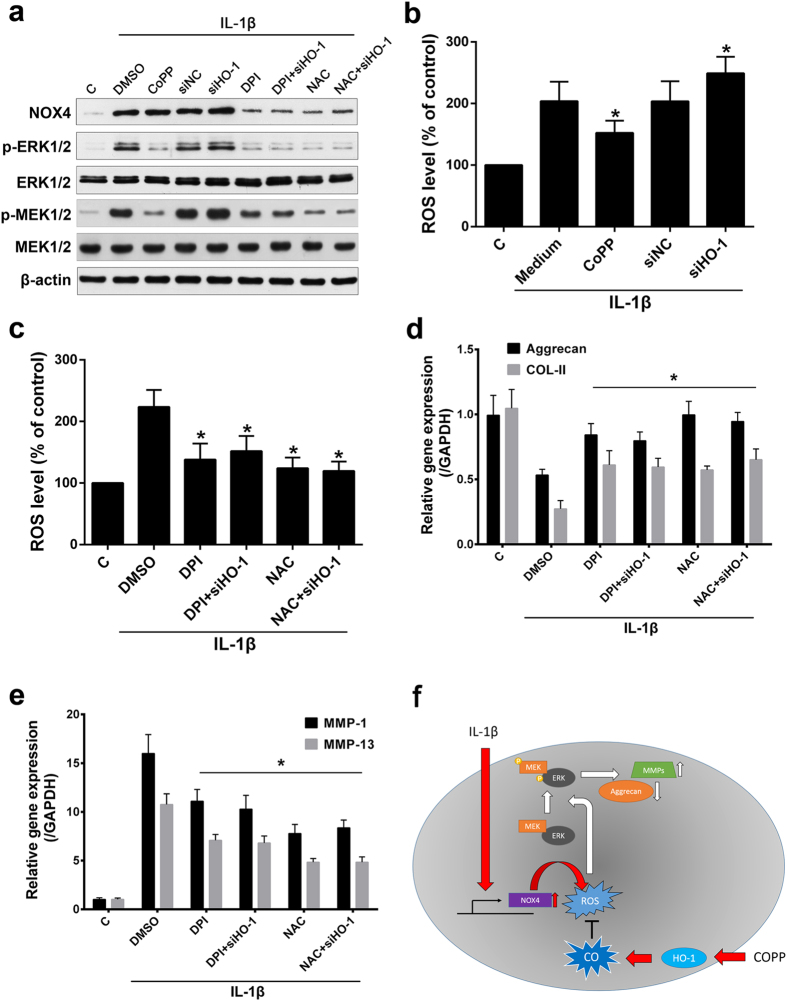
NOX/ROS regulation on the effect of HO-1 induction (**a**) Western blot analysis revealed that the increased expression of NOX4 induced by IL-1β was ameliorated by DPI or NAC, but not CoPP treatment; the IL-1β dependent activation of MEK1/2 and ERK1/2 was remarkably lowered by DPI, NAC or CoPP treatment; However, HO-1 siRNA did not alter the effect of DPI or NAC on NOX4 protein expression. (**b**) Induction of HO-1 decreased, while HO-1 knockdown increased, the elevated ROS level which stimulated by IL-1β. (**c**) Treatment of DPI or NAC substantially attenuated the increased ROS level which induced by IL-1β. (**d,e**) DPI or NAC treatment considerably weakened the effect of IL-1β on the down regulation of anabolic genes (aggrecan and COL-II) and up regulation of catabolic genes (MMP-1 and MMP-13) expression. (**f**) A summary of the mechanism of COPP in regulating MEK/ERK signaling and its downstream ECM catabolic effectors through ROS neutralizing. Values represent the mean and SD. *P < 0.05 with respect to IL-1β + DMSO (0.1%).

To further identify whether NOX/ROS system play a role in the mechanism underlying the effect of HO-1 induction, NP cells were treated with CoPP (10 μM), diphenyleneiodonium (DPI) which is a NOX inhibitor (10 μM), or with N-Acetyl-L-cysteine (NAC) which is a ROS scavenger (10 mM), in the presence or absence of IL-1β. The data revealed that IL-1β induced increment of NOX4 protein level was weakened by DPI or NAC, but not by CoPP treatment (Fig. 7a), and the activation of MEK/ERK signaling by IL-1β stimulation was considerably attenuated by DPI, NAC or CoPP treatment (Fig. 7a). Moreover, the elevated ROS level by IL-1β induction was remarkably decreased by treatment of CoPP, DPI or NAC (Fig. 7b,c). In addition, the effect of IL-1β on the reduction of anabolic genes (aggrecan and COL-II) and increment of catabolic genes (MMP-1 and MMP-13) expression was also reversed by application of DPI or NAC (Fig. 7d,e). However, HO-1 siRNA treatment did not influence the effect of DPI and NAC on MEK/ERK activation and ECM metabolic genes expression, in the presence of IL-1β (Fig. 7a,c–e). In conclusion, our data indicate that induction of HO-1 *in vitro* could attenuate the MEK/ERK signaling, thus inhibiting ECM catabolism and promote ECM anabolic activities under pro-inflammatory conditions. And the underlying mechanism of COPP in regulating MEK/ERK signaling is through neutralizing ROS by increasing CO amount during heme breaks down (Fig. 7f).

## Discussion

The imbalance of ECM metabolism in NP tissue is the pivotal pathogenic process of IDD, suggesting a loss of anabolic abilities and increment of catabolic activities^2^. Recent studies showed that the pro-inflammatory cytokine IL-1β plays a central role in the metabolic imbalance of ECM in IDD^10^,^27^,^28^. A greater understanding of this pathological process might shed light on novel therapeutic options to inhibit IDD progression. Consistent with previous studies^6^,^29^, we here show that expression of COL-II and MMP-1 is remarkably decreased and increased, respectively, in severe IDD compared to mild IDD human NP samples. Meanwhile, we show here for the first time that the expression of HO-1 in NP from patients with severe IDD is considerably decreased compared with that in NP from patients with mild IDD, and it negatively correlates with the Pfirrmann grade. In addition, to our knowledge, this is the first study to demonstrate that HO-1 plays a protective role in human NP cell ECM metabolism.

In the clinic, loss of T2-weighted signal, which reflects water content, in magnetic resonance imaging (MRI) of an intervertebral disc is thought to be the diagnostic criterion of IDD^30^. In the intervertebral disc ECM, sGAG, which carries a lot of anionic charge, is thought to be the major water-binding molecule and has been found to be severely decreased in IDD^31^. Recently, investigators revealed that IL-1β could directly impair the anabolic activity of sGAG and this was considered to be responsible for IDD progression^32^. Here, we show that induction of HO-1 can attenuate the reduction of sGAG and CS synthesis caused by IL-1β stimulation without affecting the basal level of these molecules, suggesting that HO-1 might help to maintain the ability of the intervertebral disc to retain water during inflammatory conditions in IDD.

With regard to the anabolism of major structural molecules of the ECM, loss of aggrecan and COL-II could result in the vitiation of normal disc structure and mechanical properties^2^. We demonstrate here that this process can be attenuated by HO-1 induction by CoPP without affecting their basal mRNA expression. Guillen and colleagues^15^ previously reported that HO-1 induction could enhance the basal mRNA levels of aggrecan and COL-II in chondrocytes, indicating a difference in cellular response between chondrocytes and NP cells.

SOX-6 and SOX-9 are potent positive regulators of COL-II^33^,^34^. A previous study demonstrated that IL-1β could remarkably downregulate the gene expression of SOX-6 and slightly inhibits SOX-9 expression in human NP cells^10^. In agreement with this study, we observed that IL-1β led to a conspicuous decrease of SOX-6 gene expression in human NP cells. Notably, our results show that gene expression of SOX-9 is not modulated by IL-1β, and we also show that downregulation of SOX-6 gene expression can be rescued by HO-1 induction. Combining the above, these results suggest that downregulation of HO-1 is associated with IDD progression, and that induction of HO-1 might protect the anabolism of NP from pernicious effects of pro-inflammatory cytokines.

Concerning the excessive catabolic activities in IDD, the role of the degrading enzymes MMPs and ADAMTs in ECM catabolism in NP has been addressed by other investigators^8^,^9^, and there is convincing evidence that the pro-inflammatory cytokine IL-1β participates in the modulation of these enzymes^10^,^25^,^28^. MMP-1 and MMP-13 are the major collagenases which cause degradation of COL-II in the ECM of NP^29^,^35^; MMP-3 is the stromelysin which degrades both COL-II and proteoglycans^36^; MMP-9 is the gelatinase which participates in the degradation of gelatin and aggrecan in the intervertebral disc^37^, and ADAMTS-4 and ADAMTS-5 are the aggrecanases which mainly function to reduce aggrecan content^38^. The expression of all these enzymes, which are often seen as the catabolic markers of IDD, has been reported to be increased in degenerated human discs^29^,^35^,^36^,^37^,^38^. We here demonstrate that the expression of all these catabolic enzymes is upregulated by IL-1β treatment as expected, and that induction of HO-1 reverses the effects of IL-1β on these molecules, suggesting that HO-1 induction not only protects anabolism, but also prevents the catabolic process in human NP from pro-inflammatory cytokine stimulation.

Intriguingly, we observed that CoPP did not affect the basal mRNA levels of MMP-1, MMP-3, MMP-9 and MMP-13, whereas it resulted in remarkably decreased basal protein levels of these molecules. These results suggest that the HO-1 regulation of these MMPs might be an independent cellular mechanism from its modulation of IL-1β responses, implicating a pivotal role of HO-1 induction against NP degradation.

With regard to the underlying mechanism of the effects of HO-1 induction, previous studies reported that IL-1β activates several major cellular signaling pathways in NP cells, including nuclear factor-κB and MAPK^25^,^39^. In addition, investigators have revealed that activation of MAPK signaling by IL-1β results in impaired function of ECM anabolism and catabolism in intervertebral discs^25^,^40^. Xia and colleagues^21^ demonstrated that ERK1/2 modulates the expression of COL-II, MMP-9 and MMP-13 in human NP cells; ERK1/2 signaling has also been reported to be involved in the regulation of aggrecan, MMP-3, and ADAMTS-4 expression in NP cells under IL-1β stimulation^25^,^41^, and there are also evidences suggested that ERK1/2 was the upstream regulator of MMP-1, SOX-6 and ADAMTS-5^42^,^43^. We here show that HO-1 remarkably inhibits activation of ERK1/2, and shows no effect to p38 and JNK activation caused by IL-1β stimulation. To further ascertain the concrete mechanism of effects of HO-1 induction, we tested whether CoPP affected the upstream regulators of ERK1/2, namely MEK1/2 and Ras^44^, which have also been tested. The data show that CoPP attenuates the activation of MEK1/2 by IL-1β, while the alteration of Ras level cannot be detected, this suggest that inhibition of MEK/ERK signaling by CoPP under IL-1β stimulation may contribute to its effect against with IL-1β. We also show that the expressions of downstream effectors of ERK1/2, CDK1, CCL3 and CCND1^44^,^45^,^46^, were reversed by CoPP treatment under IL-1β stimulation, while the level of downstream molecules of JNK (GADD45, Kip1 and Bim)^47^ and p38 (FOXM1, E2F1 and p62)^48^,^49^ has not been affected. Taken together, these findings suggest that inhibition of MEK/ERK signaling by HO-1 induction, might contribute to the alleviation of ECM metabolic disorder.

Previous studies demonstrated that NOX4 and ROS involved in the regulation of HO-1 on ECM degrading enzymes in chondrocytes^16^,^50^. Since NP cells possess a similar phenotype with chondrocytes, we tested whether NOX/ROS system also participant in the regulatory process in NP cells. Consistent with previous findings, we show here that HO-1 induction could abate the ROS production which induced by IL-1β. Moreover, inhibition of NOX/ROS result in lower activation of MEK/ERK signaling which activated by IL-1β. However, knockdown of HO-1 by siRNA could not alter the inhibitory effect of ROS scavenger NAC and NOX inhibitor DPI on MEK/ERK activation, this suggest that HO-1 may not involve in the downstream of NOX/ROS regulation on MEK/ERK signaling. NOX/ROS inhibition also reverses the expression of ECM catabolic and anabolic markers which induced by IL-1β. Taken together, the HO-1 regulation on NOX/ROS system may play a pivotal role in its effect on MEK/ERK signaling and ECM metabolism, which in the presence of IL-1β.

Heme oxygenase-1 (HO-1) breaks down heme to biliverdin, CO and ferrous iron^13^. Previously, investigators have demonstrated that treatment of carbon monoxide releasing molecule (CORM), a CO donor, could decrease ROS production and MMP-1 expression in chondrocytes which activated by IL-1β, while a control molecule ruthenium III chloride (RuCl) show no effect on this^16^. We first show here that CO attenuates the effect of IL-1β on ROS production, ECM anabolic and catabolic genes expression in nucleus pulposus cells, this suggest CO production which induced by HO-1 may also play a role in its anti-inflammatory mechanism (See Supplementary Figure S1).

However, our study has some drawbacks. First, we used mild degenerated NP tissues (Pfirrmann grade II/III) in place of non-degenerated tissues as the control group. Currently, no diagnostic gold standard exists for IDD and some researchers have indicated that IDD occurs naturally with age^51^,^52^. Therefore, it is difficult to identify whether disc samples are non-degenerated. However, based on our grouping methods, we did observe some differences between the mild and severe IDD group. Next, although we used real-time PCR and ELISA to examine the expression of MMPs at mRNA and protein level in human NP cells, the actual enzymatic activities of MMPs could not be assessed by our methods. However, MMPs are regarded as the catabolic marker and the downstream indicators of ECM catabolism in intervertebral discs, which have been addressed by many studies^29^,^35^,^36^,^37^, so it is not unreasonable to assume that the data we present might indicate a counteracting role of HO-1 on the induction of MMPs by IL-1β in human NP cells.

In conclusion, our data indicate that HO-1 might play a pivotal role in IDD. Induction of HO-1 *in vitro* could attenuate the increase in ECM catabolism and promote ECM anabolic activities under pro-inflammatory conditions. These findings suggest that manipulating HO-1 expression in the intervertebral disc might mitigate the impairment of ECM metabolism resulting from pro-inflammatory cytokine stimulation, and thus could offer a novel therapeutic approach to the treatment of IDD.

## Methods

### Collection and grading of human tissue samples

Informed consent was given by the patients or relatives to obtain human intervertebral tissue at surgery. The experimental methods were carried out in accordance with the approved guidelines and the study was authorized by the ethics committee of Second Military Medical University. Forty nucleus pulposus samples with different degrees of IDD (n = 40; age 25 to 78 years, mean age 46.5 years) were obtained from patients (See Supplementary Table S1), who underwent disc resection surgery or spinal fusion to relieve LBP. Patients diagnosed with classical sciatica were excluded from the experiment. MRI T-2 weighted images were collected and the modified Pfirrmann grading system^53^ was used to evaluate the degree of IDD. In this study, samples of grade-II (G-II) and grade-III (G-III) were set as the mild IDD group; grade-IV (G-IV) and grade-V (G-V) samples were set as the severe IDD group.

### Isolation of nucleus pulposus cells

For cell extraction, NP tissue specimens were washed twice with PBS, then minced and digested with 2 U/mL protease in DMEM/F12 medium (Gibco, Grand Island, NY, USA) for 30 minutes at 37 °C. NP cells were released from the NP tissues by treating with 0.25 mg/mLtype II collagenase (Gibco, Cat. No. 17101-015) for 4 hours at 37 °C. The remaining cell suspension was transferred into a 40 μm cell strainer (BD Biosciences, Franklin Lakes, NJ, USA) and centrifuged at 800 g for 5 minutes. The NP cells were resuspended in DMEM/F12 containing 10% FBS (Gibco), 100 U/mL penicillin, 100 μg/mL streptomycin, and 1% L-glutamine. The viability of the suspended cells was over 90% when assessed using cell counting kit-8 (Dojindo, Tokyo, Japan). Cells were incubated at 37 °C in 5% CO_2_ and the medium was changed every 3 days. Cells at the second passage were used for subsequent experimental procedures.

### Nucleus pulposus cell culture and treatment

When the NP cells reached confluence, the cells were trypsinized with 0.25% trypsin/ethylenediaminetetraacetic acid (Gibco) and seeded into 6/24-well plates at 150,000/10,000 cells/well in the same medium. When cells in the plates reached confluence, we incubated the cells with 10 μM CoPP (Frontier scientific, Logan, UT, USA), 10 μM DPI, 10 mM NAC, 100 μM CORM-3 or with 100 μM RuCl (Sigma-Aldrich, St Louis, MO, USA) for 1 h before activation with 10 ng/mL IL-1β (Peprotech, Rocky Hill, NJ, USA) for different times.

### Si-RNA transfection

Pre-designed si-RNA and negative control si-RNA to silence the human HO-1 gene were purchased from Ambion (Foster City, CA, USA). For transfection, cells were seeded into 6-well plates, incubated for 24 h, then transfected with HO-1 si-RNA (100 nM), or negative control si-RNA, using Lipofectamine® RNAiMAX Transfection Reagent (Invitrogen, Carlsbad, CA, USA) according to the manufacturer’s instructions. After subsequent treatments, cells were harvested for analysis.

### RNA extraction and reverse transcription

Human NP cells were activated with IL-1β (10 ng/mL) in presence or absence of inhibitors (CoPP, 10 μM; DPI, 10 μM; NAC, 10 mM) for 24 h. For experiments with application of CORM-3 (100 μM) or RuCl (100 μM), the treatment time duration was 16 h. RNA was extracted from human nucleus pulposus samples using Trizol (Invitrogen) according to the manufacturer’s instructions. The concentration of total RNA was measured at 260 nm with a spectrophotometer (DU-800; Beckman Coulter, Brea, CA, USA). First strand complementary DNA (cDNA) synthesis was performed with 500 ng of total RNA in a 10μLfinal volume containing 2 μL PrimerScript RT Master Mix (RR036A, Takara Bio Inc., Shiga, Japan) and 8μLof RNase-Free dH_2_O and total RNA. The reverse transcription procedure was carried out according to the manufacturer’s instructions.

### Quantitative real-time polymerase chain reaction

Real-time PCR for glyceraldehyde-3-phosphate dehydrogenase (GAPDH), HO-1, aggrecan, COL-II, SOX-6, SOX-9, MMP-1, MMP-3, MMP-9, MMP-13, ADAMTS-4, ADAMTS-5, CDK1, CCL3, CCND1, GADD45, Kip1, Bim, FOXM1, E2F1 and p62 was performed using SYBR premix Ex Taq™ (Takara) with a Step One Plus real-time PCR system (Applied biosystems, Foster City, CA, USA), according to the manufacturer’s instructions. GAPDH was used to normalize the gene expression of other mRNAs. The relative amount of each transcript was calculated according to the comparative Ct method. All of the primers were synthesized by Invitrogen (Life technologies) (See Supplementary Table S2).

### Immunohistochemical and image analysis procedure

Immunohistochemistry was performed to localize HO-1, COL-II, and MMP-1 in 7 NP samples of Pfirrmann grade II, 13 NP samples of grade III, 15 NP samples of grade IV, and 5 NP samples of grade V. The general immunohistochemistry protocol has been described elsewhere^54^. Briefly, antigen retrieval was performed using trypsin for 30 min at 37 °C, and the sections were blocked with 1% bovine serum albumin for 15 min at room temperature. Next, the sections were incubated at 4 °C overnight with the following primary antibodies: rabbit polyclonal antibody against HO-1 (1:100 dilution), rabbit polyclonal antibody against COL-II (1:100 dilution), and rabbit polyclonal antibody against MMP-1 (1:100 dilution) (Proteintech, Chicago, IL, USA). Next, the secondary antibody peroxidase-conjugated affinipure goat anti-rabbit IgG (1:1000 dilution) (Proteintech) was applied to the sections and they were counterstained with hematoxylin. The image analysis procedure has been previously described^55^, briefly, samples were imaged using a ZEISS microscope (ZEISS Axio Imager A2, Carl Zeiss microscopy GmbH, Jena, Germany). Immunostained slides were identified independently by three pathologists who were blinded to patients’ data and outcomes. When there were different evaluation results, a consensus result should be achieved after reexamination. Within each sample, 200 cells were counted and the number of immunopositive cells expressed as a proportion of this.

### DMMB assay

Human NP cells were treated with IL-1β (10 ng/mL) or IL-1β + CoPP (10 μM) for 6 days. Then, the DMMB assay was performed using a Blyscan™ sGAG Assay kit (B1000, Biocolor Ltd., Carrickfergus, UK) according to the manufacturer’s instructions. Briefly, harvested NP cells were washed with PBS then digested with 1mLpapain extraction reagent for 24 hours at 65 °C. The sGAG content was determined by reaction with DMMB, and staining was quantified by measuring absorbance at 656 nm. Chondroitin-4-sulfate was used as the standard.

### Immunofluorescence microscopy

Human NP cells were plated into 24-well plates and treated with IL-1β (10 ng/mL) or IL-1β + CoPP (10 μM) for 72 h. After incubation, the cells were fixed with 4% paraformaldehyde and permeabilized with 0.3% Triton X-100 in PBS for 10 min. Next, the cells were blocked with 5% bovine serum albumin in PBS and incubated with a mouse monoclonal antibody against chondroitin sulfate (1:200 dilution) (Sigma-Aldrich, St Louis, MO, USA; catalog no. C8035) at 4 °C overnight. The cells were washed with PBS and incubated with the secondary antibody goat anti-mouse IgG-FITC (1:128 dilution) (Sigma-Aldrich; catalog no. F5262) for 1 h at room temperature. Samples were imaged using a fluorescence microscope (ZEISS Axio Imager A2, Carl Zeiss microscopy GmbH, Jena, Germany). As previously described^56^,^57^, within each sample, eight different visual fields, with at least 20 cells in each visual field were randomly selected, and a region was drawn around each cell to be measured, the same size region was drawn in an area without fluorescence objects to be utilized for background subtraction, and the average relative fluorescence intensity of each cell was analyzed using the image J software (NHI, Bethesda, MD, USA).

### Enzyme-linked immunosorbent assay

NP cells were activated with IL-1β (10 ng/mL) or IL-1β + CoPP (10 μM) for 48 h. Supernatants were harvested, centrifuged and frozen at −80 °C until analysis. Levels of MMP-1, 3, 9, and 13 proteins were measured using an ELISA kit (BosterBio, Pleasanton, CA, USA) according to the manufacturer’s instructions. The results were normalized to the total protein concentration.

### Western blot analysis

Human NP cells were harvested using iced-cold lysis buffer (Cell Signaling Technology, Danvers, MA, USA). For detection of phosphorylation proteins level, the total protein was extracted at 15 min after treatment, while the other treatment duration was 24 h. Protein concentration was determined using a bicinchoninic acid protein assay kit (Pierce Biotechnology, Rockford, IL, USA). Immunolabeling was detected using the Pierce ECL western blotting substrate (Pierce Biotechnology, Rockford, IL, USA). The following antibodies and dilutions were used: HO-1 antibody (1:500) (Proteintech), β-actin (1:1000) (Santa Cruz Biotechnology, Santa Cruz, CA, USA); phospho-ERK1/2 (1:1000), ERK1/2 (1:1000), phospho-p38 (Thr180/Tyr182) (1:1000), p38 (1:1000), phospho-JNK (T183/Y185) (1:1000), JNK (1:1000), phospho-MEK1/2 (1:1000), MEK1/2 (1:1000) and Ras (1:1000) (Cell Signaling Technology).

### Immunocytochemistry

Human NP cells were seeded into 24-well plates and allowed to expand to near confluence. At this point, cells were incubated with CoPP (10 μM) in the presence or absence of IL-1β (10 ng/mL) for 15 days, with replacement of medium and treatment every 4 days. Cells were fixed with 4% formaldehyde in PBS for 30 min at 4 °C and rabbit anti-collagen type II polyclonal antibody (1:100 dilution) (Proteintech) was used to examine the expression of collagen type II. Peroxidase-conjugated affinipure goat anti-rabbit IgG (1:1000 dilution) (Proteintech) was used as the secondary antibody.

### Assessment of the level of Reactive Oxygen Species

Cells were seeded in a 96-well plate and treated with IL-1β (10 ng/mL) in presence or absence of inhibitors (CoPP, 10 μM; DPI, 10 μM; NAC, 10 mM) (Sigma-Aldrich, St Louis, MO, USA) for 24 h. CORM-3 (100 μM) or RuCl (100 μM) was introduced 1 h before ROS measurement started. ROS generation was detected by using 2′, 7′-Dichlorodihydrofluorescein diacetate (DCFH-DA, Sigma-Aldrich) as previously described^58^, DCFH-DA is oxidized by ROS, into the fluorescent dichlorofluorescein (DCF). For quantification of ROS level, the treated cells were stained with DCFH-DA for 30 min at 37 °C, assessments (excitation: 485 nm/emission 520 nm) were performed by using a microplate reader spectrophotometer (Infinite M200, Tecan, Austria) as manufacturer’s instructions. The results were normalized to the total protein concentration, and ROS level was presented as a% ration of the control group.

### Statistical analysis

Data are presented as mean ± standard deviation of at least three independent experiments. GraphPad Prism 6.0 software (GraphPad Software, Inc., San Diego, CA, USA) was used for statistical analysis. The normality of the data was tested using the D’Agostino-Pearson omnibus normality test. The data of the proportions of immunopositive NP cells in human NP tissues did not pass the normality test; therefore these data were analyzed by applying the two-tailed Mann-Whitney U test. The remaining data passed the normality test, and the two-tailed Student’s t-test or an analysis of variance (ANOVA) followed by the Turkey’s t-test were performed for comparison of two groups or multiple groups, respectively. The significance threshold was 0.05.

## Additional Information

**How to cite this article**: Hu, B. *et al.* Heme oxygenase-1 attenuates IL-1β induced alteration of anabolic and catabolic activities in intervertebral disc degeneration. *Sci. Rep.* **6**, 21190; doi: 10.1038/srep21190 (2016).

## Supplementary Material

Supplementary Information
