# Supplementary material for: Heme oxygenase-1 attenuates IL-1β induced alteration of anabolic and catabolic activities in intervertebral disc degeneration
Source: Sci Rep. 2016 Feb 15;6:21190. doi: 10.1038/srep21190 (PMC4753421; doi:10.1038/srep21190)
Supplement: Supplementary Information [file srep21190-s1.pdf]

Heme oxygenase-1 attenuates IL-1β induced alteration of anabolic and catabolic activities in intervertebral disc degeneration

Bo Hu[1†], Changgui Shi[1†], Chen Xu[1†], Peng Cao[1], Ye Tian[1], Ying Zhang[1], Lianfu Deng[2], Huajiang Chen[1*], Wen Yuan[1*]

[1]Department of Spinal Surgery, Changzheng Hospital, Second Military Medical University, No. 415 Feng Yang Rd, Shanghai 200003, China

[2]Shanghai Key Laboratory for Bone and Joint Diseases, Shanghai Institute of Orthopaedics and Traumatology, Shanghai Ruijin Hospital, Shanghai Jiao Tong University School of Medicine, China.

[*]Corresponding authors:

Huajiang Chen; E-mail: spine.chen@163.com

Department of Spinal Surgery, Changzheng Hospital, Second Military Medical University, No. 415 Feng Yang Rd, Shanghai 200003, China

Wen Yuan; E-mail: yuanwenspine@126.com

Department of Spinal Surgery, Changzheng Hospital, Second Military Medical University, No. 415 Feng Yang Rd, Shanghai 200003, China

[†]Bo Hu, Changgui Shi and Chen Xu are equally contributed to this paper.

Supplementary Table S1. Clinical findings in 40 patients with IDD

laboratory number	age	sex	disc level	Duration of Symptoms (mo)	pfirrmann grade
1	25	F	L5/S1	23	2
2	30	M	L5/S1	13	2
3	35	F	L4/5	4	2
4	28	M	L4/5	6	2
5	27	M	L5/S1	17	2
6	25	F	L3/4	12	2
7	32	M	L4/5	12	2
8	46	F	L5/S1	26	3
9	33	F	L3/4	22	3
10	60	F	L4/5	6	3
11	57	F	L4/5	24	3
12	58	M	L5/S1	12	3
13	48	F	L4/5	16	3
14	37	F	L5/S1	9	3
15	52	M	L4/5	17	3
16	53	M	L5/S1	18	3
17	37	M	L4/5	6	3
18	53	F	L5/S1	14	3
19	41	F	L4/5	24	3
20	39	M	L4/5	16	3
21	78	F	L5/S1	33	4
22	53	F	L5/S1	12	4
23	41	M	L5/S1	29	4
24	42	F	L5/S1	16	4
25	66	F	L4/5	11	4
26	46	M	L5/S1	23	4
27	38	M	L5/S1	15	4
28	69	F	L4/5	26	4
29	40	F	L4/5	12	4
30	44	F	L5/S1	24	4
31	50	M	L4/5	6	4
32	42	M	L4/5	16	4
33	57	F	L5/S1	19	4
34	47	F	L5/S1	18	4
35	51	F	L4/5	15	4
36	42	M	L4/5	7	5
37	56	M	L4/5	21	5
38	53	M	L3/4	11	5
39	61	F	L4/5	19	5
40	67	F	L5/S1	28	5

Supplementary Table S2. Primer sequences

Gene	Forward (5’→3’)	Reverse (5’→3’)
HO-1	CAGGCAGAGAATGCTGAGTTC	GCTTCACATAGCGCTGCA
COL-II	GGCAATAGCAGGTTCACGTACA	CGATAACAGTCTTGCCCCACTT
Aggrecan	TCGAGGACAGCGAGGCC	TCGAGGGTGTAGCGTGTAGAGA
SOX-6	CCGTGAGATAATGACCAGTGTTACTT	GTCCACCACATCGGCAAGAC
SOX-9	GACTTCCGCGACGTGGAC	GTTGGGCGGCAGGTACTG
MMP-1	GGGGCTTTGATGTACCCTAGC	TGTCACACGCTTTTGGGGTTT
MMP-3	CGGTTCCGCCTGTCTCAAG	CGCCAAAAGTGCCTGTCTT
MMP-9	CCCGGAGTGAGTTGAACCA	TACGTGACCTATGACATC
MMP-13	GGACAAGTAGTTCCAAAGGCTACAA	CTTTTGCCGGTGTAGGTGTAGATAG
ADAMTS-4	ACTGGTGGTGGCAGATGACA	TCACTGTTAGCAGGTAGCGCTTT
ADAMTS-5	GGACCTACCACGAAAGCAGATC	GCCGGGACACACGGAGTA
CDK1	AAACTACAGGTCAAGTGGTAGCC	TCCTGCATAAGCACATCCTGA
CCL3	AGTTCTCTGCATCACTTGCTG	CGGCTTCGCTTGGTTAGGAA
CCND1	TGGAGCCCGTGAAAAAGAGC	TCTCCTTCATCTTAGAGGCCAC
GADD45	GCACGCAGCCTACTAGGTG	CGAACTGCTTAGCCGCGTA
Kip1	AACGTGCGAGTGTCTAACGG	CCCTCTAGGGGTTTGTGATTCT
Bim	TAAGTTCTGAGTGTGACCGAGA	GCTCTGTCTGTAGGGAGGTAGG
FOXM1	CGTCGGCCACTGATTCTCAAA	GGCAGGGGATCTCTTAGGTTC
E2F1	ACGCTATGAGACCTCACTGAA	TCCTGGGTCAACCCCTCAAG
p62	CACACCCTCTCTACTCGGG	ACATGCCAGGTAATAGGCTTTC
GAPDH	CCATGTTCGTCATGGGTGTGAACCA	GCCAGTAGAGGCAGGGATGATGTTC

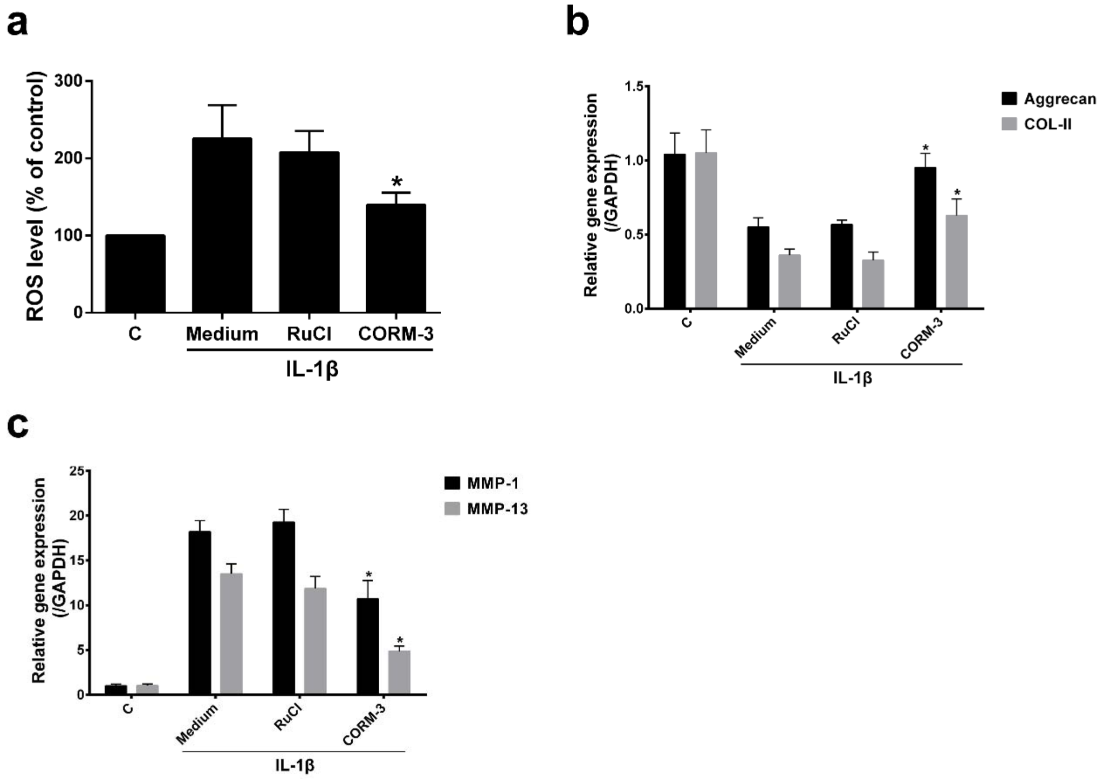

Supplementary Figure S1. Carbon monoxide ameliorated the effect of IL-1β on ROS production and ECM metabolic genes expression

(**a**) CORM-3 treatment remarkably reduced the ROS level which increased by IL-1β, while control molecule RuCl fail to show any effect on ROS production. (**b, c**) Treatment of CORM-3 attenuated the effect of IL-1β on the reduction of anabolic genes (aggrecan and COL-II) and enhancement of catabolic genes (MMP-1 and MMP-13) expression. Values represent the mean and SD. **P*<0.05 with respect to IL-1β + Medium.
